# Future food prices will become less sensitive to agricultural market prices and mitigation costs

**DOI:** 10.1038/s43016-024-01099-3

**Published:** 2025-01-03

**Authors:** David Meng-Chuen Chen, Benjamin Bodirsky, Xiaoxi Wang, Jiaqi Xuan, Jan Philipp Dietrich, Alexander Popp, Hermann Lotze-Campen

**Affiliations:** 1 Potsdam Institute for Climate Impact Research (PIK), Member of the Leibniz Association, Potsdam, Germany; 2 Humboldt-Universität zu Berlin, Faculty of Life Sciences, Thaer-Institute for Agricultural and Horticultural Sciences, Berlin, Germany; 3 Humboldt-Universität zu Berlin, Integrative Research Institute on Transformations of Human-Environment Systems (IRI THESys), Berlin, Germany; 4 China Academy for Rural Development, School of Public Affairs, Zhejiang University, Hangzhou, China; 5 Faculty of Organic Agricultural Sciences, University of Kassel, Witzenhausen, Germany

**Keywords:** Environmental studies, Economics, Development studies

## Abstract

Agricultural production costs represent less than half of total food prices for higher-income countries and will likely further decrease globally. Added-value components such as transport, processing, marketing and catering show increasing importance in food value chains, especially as countries undergo a nutrition transition towards more complex and industrial food systems. Here, using a combined statistical and process-based modelling framework, we derive and project the value-added component of food prices for 136 countries and 11 different food groups, for food-at-home and food-away-from-home. We identify the declining but differentiated producer share in consumer food prices across food products, and provide scenarios of future consumer prices under a business-as-usual as well as climate mitigation scenarios. Food price increases from policies targeting agricultural producers, such as greenhouse gas taxes, are not as stark when transmitted to consumers owing to higher value added in higher-income countries, while a pronounced effect remains in lower-income countries, even in coming decades.

## Abstract (web summary)

The future of food prices is uncertain yet key for food security and climate mitigation policies. This study estimates future food prices for 136 countries and 11 distinct food groups, showing that future food prices will become less sensitive to agricultural market dynamics and land-based mitigation policies, given the global transition towards more complex and industrial food systems.

## Main

Under the ongoing global nutrition transition, people consume more animal products, more highly processed foods and more food-away-from-home (FAFH)^1,2^. Food systems become more complex, with the food processing and food service sectors taking up a large portion of consumer food spending; the agricultural producer’s share in final food expenditures (‘farm share of the food dollar’ or ‘farm share’) thus decreases. For example, the farm share of a dollar spent on food in the United States has steadily declined, estimated at almost 50% in the 1950s to below 20% in 2017^3^.

Lower-income countries will likely also follow such trajectories^3,4^, given currently estimated trajectories of income and consumption. With economic development, consumers allocate an increasing absolute spending on food, while relative share of food as total income decreases. Additional food expenditures in higher-income countries go to animal-based products, luxury and processed foods, and other non-staple foods; in low-income countries, a higher share of household spending is dedicated to food, most of which goes to staples^5^. As people consume an increasing share of FAFH as incomes increase, this leads to even higher value-added shares. About 55% of consumer food expenditures were spent on FAFH in the United States in 2021^6^, and in Korea, 49% of calories consumed stemmed from FAFH^1^. In China, where economic development has driven a strong transition in its food system, 26% of calories consumed there in 2016 stemmed from FAFH^7,8^. As such, consumer food prices are the product of multiple food value chain (FVC) components besides agricultural production costs, including transport, processing, marketing, retail and catering services^9^.

There is limited empirical evidence on how the marketing margin (the difference between consumer and producer prices) changes over time and with income^10^, with some indication that distribution margins (post-farmgate margins but not including transport) increase with income, but only for richer countries^11^. However, as lower-income countries develop economically, they may also begin to experience increasing margins as part of the nutrition transition^12^. As such, understanding the future development of value-added components of FVCs becomes of increasing importance for sustainability, health and economic inclusion assessments of the food system^4,12,13^.

Increased demand for processed and especially animal-based foods is also a large contributor to anthropogenic climate change^14,15^. Greenhouse gas (GHG) emissions from the food and land system must fall drastically in the next decades to meet globally negotiated policy goals such as the Paris Accord. Along with deeper systemic changes, policies such as GHG prices are necessary for effective and efficient reductions of food system emissions in a timely manner^16^. However, especially as consumer demand shifts towards higher-emissions foods, recent studies have highlighted that such policies can potentially lead to increased agricultural commodity prices, via multiple pathways such as higher marginal production costs, and increased competition for land^17,18^. It is thus important to consider potential trade-offs between land-based mitigation and food security^17,19^, especially in lower-income countries where such issues are most salient.

Consumer food prices are the product of multiple FVC components, as noted. However, global economic models that provide scenarios assessing GHG mitigation policies’ impacts on food prices typically only partially cover value-added components in the food supply chain. Two broad categories of models often applied to assess the impact of climate mitigation policies on food prices in an integrated approach are partial equilibrium (PE) and computable general equilibrium (CGE) models. In PE models, supply chain sectors beyond the first stage of processing are typically not included, with price elasticities being based on consumer expenditures^5,20^. In CGEs, there may be strong assumptions of the share of value-added processing in the consumer demand for food, or detailed representation is limited to specific world regions^12,20^. As such, assessments of trade-offs between land-based climate mitigation policies and food security may overstate the relative impact of increased agricultural production costs on consumers, especially in PE models, as future consumer food expenditures will likely increasingly be directed towards the value-added components of the food system as opposed to the agricultural production.

In this study, we assess how climate mitigation policies affect food consumers, while taking added value from FVC into account explicitly, with the aim of providing a more accurate account of how consumers may be impacted. We first generate a dataset of food marketing margins and consumer prices; in a second step, we estimate future consumer food prices under a baseline scenario and a climate mitigation scenario. The marketing margin is defined here as the difference between consumer and producer prices for an equivalent amount of primary product (that is, based on the amount of primary product embedded in consumers’ purchases), which we calculate for 11 broad food categories for 2 years (2011 and 2017) and 138 countries, for food-at-home (FAH) as well as FAFH prices. We then derive and validate a relationship between the added-value margin and average per-capita income by applying a nonlinear hierarchical Bayesian regression model.

This model allows for the estimation of FAH and FAFH consumer prices and expenditures by food product, for the past decade (2010–2019) as well as for the future, when projections of income and of producer prices are available. To derive such projections and assess how the relation between producer and consumer prices develops into the future, we use in the second step a process-based integrated food and land system modelling framework (the MAgPIE model^21^) to derive long-term producer price projections. This further allows us to test possible future policy implementations, isolating how the inclusion of GHG taxation policies to the land sector would affect consumer prices. This is of particular interest, as the AFOLU (Agriculture, Forestry and Land Use) sector and land-use change emissions are often not included in current emissions trading protocols such as the European Emissions Trading System owing to apprehension about food price increases^22^. We thus project the evolution of future consumer food prices for a business-as-usual (BAU) future as well as a scenario with an ambitious GHG mitigation policy (POL), with a carbon price trajectory on land-based mitigation that achieves the 1.5 degree warming limit.

This study thus provides an assessment of the importance of FVC components in how food consumers are affected by land-based climate mitigation policies. It also calculates indicators such as the ‘farm share’ at present and in the future, assisting the design of effective policies related to FVCs and the agricultural economy^3,12^.

## Results

**Fig. 1:**
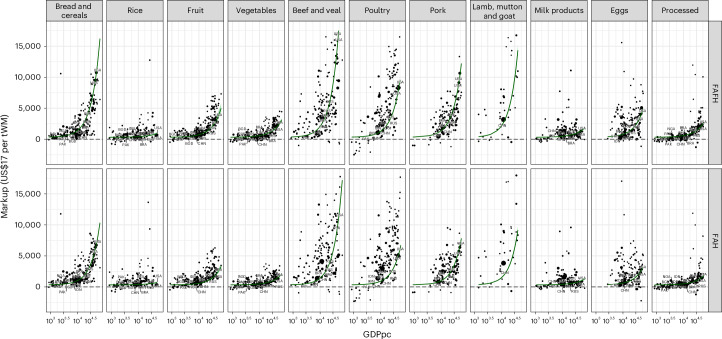
Consumer price markups by price and consumption. FAH and FAFH consumer price marketing margins (difference between final food consumer price and agricultural market price) for broad food group categories. Points are country-year combinations for years 2011 and 2017; point size denotes country population. Marketing margin in constant 2017 USD per ton wet matter primary product, *x*-axis per capita GDP in constant 2017 USD (GDPpc), on a log scale for better visualization of lower-income countries. The green line (centre value) is the line of best fit, using nonlinear exponential Bayesian regression, light green shading the 95% CI. Bayes-R2 = 0.34. PAK, Pakistan; BGD, Bangladesh; NGA, Nigeria; IDN, Indonesia; CHN, China; BRA, Brazil; RUS, Russia; USA, United States of America.

In Fig. 1, we show the marketing margins derived across the 11 food groups and location (FAH and FAFH) of consumption, for both 2011 and 2017, in relation to each country’s per-capita income of that year. The marketing margins range from close to zero to greater than 10,000 US dollars per ton of primary agricultural product (wet matter basis). Importantly, we calculate the marketing margin based on a difference as opposed to a ratio, given the assumption that added value stems from processes dependent mainly on physical quantities: transport, processing, packaging and marketing are generally value-added per physical unit of agricultural commodity, as opposed to per dollar producer price.

We observe that the difference between consumer food prices and producer prices exhibits nonlinear dynamics as incomes rise (see Supplementary Fig. 1 for a non-log scale). As such, an exponential function provides a good fit, along with the flexibility to fit trends to all product categories. Furthermore, the functional form selected can also be interpreted as an approximation of a percentage change relationship, that is, an income elasticity. Expressed thus, we find that on average globally, for a 1% increase in income, the marketing margins increase by 1.38% (95% credible interval (CI) of 1.35, 1.40 CI). Note that this value is more than unit elastic, that is, a 1% change in per-capita GDP leads to more than 1% change in the margin; the margin thus increases at an increasing rate. The various meat as well as the various ‘bread and cereals’ as well as animal-based food categories see the highest income elasticities of 1.4% and above, although this ranges for the various meat products (Supplementary Table 1). This indicates that the value added for these products increases much faster as per-capita incomes increase. ‘Vegetables’, ‘fruit’, ‘milk products’ and ‘rice’ have relatively lower elasticities, between 1.12 and 1.26. Furthermore, the regression parameterization provides a multiplicative coefficient varying by FAH and FAFH, for which FAH is 63% that of FAFH. As such, we note that on average globally, food consumed away from home is about a third more expensive than food purchased for at-home consumption. All regressions are fit within a Bayesian framework that allows for flexible fitting of functional forms to grouped data, the partial pooling of information across groups and intuitive interpretation of uncertainties (Methods).

**Fig. 2:**
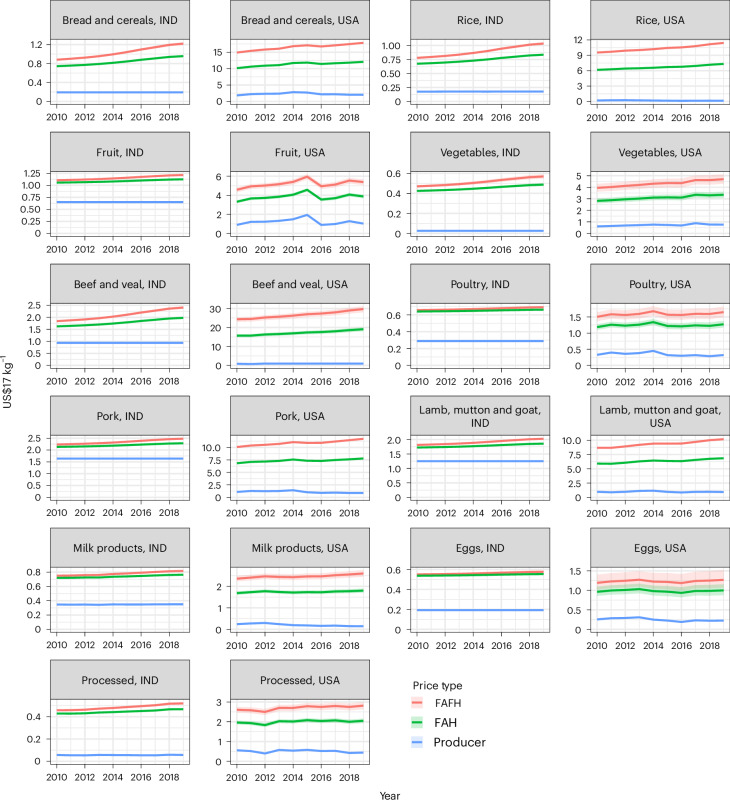
Food prices along the FVC. Producer prices and FAH and FAFH consumer food prices for USA and IND, by applying the marketing margins estimated in this study on FAOSTAT producer prices. Prices are weighted averages based on consumption quantities. Shaded area 95% CI from regression propagated.

By applying the model-estimated marketing margins to producer prices reported in FAOSTAT^23^, we can derive consumer food prices for both FAH and FAFH prices for 180 countries, for the years 2010–2019, and across the 11 aggregate food product categories extracted from the International Comparison Program (ICP). Figure 2 shows these food prices for the United States and India, as examples of two countries on the opposite sides of the nutrition transition. These results demonstrate the importance of value added in consumer prices as GDP increases. Between the United States and India, producer prices for bread and cereals in 2019 are, for example, relatively similar, at US$0.17 kg^−1^ and US$0.23 kg^−1^, respectively. However, FAH consumer prices are US$12 (11.53, 12.38) kg^−1^ in the United States and US$0.96 (0.93, 0.99) kg^−1^ in India, and FAFH prices are US$17.29 (16.69, 18.24) kg^−1^ in the United States and only US$1.22 (1.18, 1.26) kg^−1^ in India. We note that in the United States and other high-income countries, producer prices constitute a very small fraction of the final price, especially when considering out-of-house consumption. For ‘bread and cereals’, the producer price is less than 2% of the consumer FAFH price in the United States in 2019, while it is 20% in India. Similarly, for ‘eggs’, which has the smallest marketing margin and least processing from farm to table in India, this share is 58% in India, while the smallest marketing margin share by product in the United States is for ‘vegetables’, where the producer price is 28% that of FAFH.

**Fig. 3:**
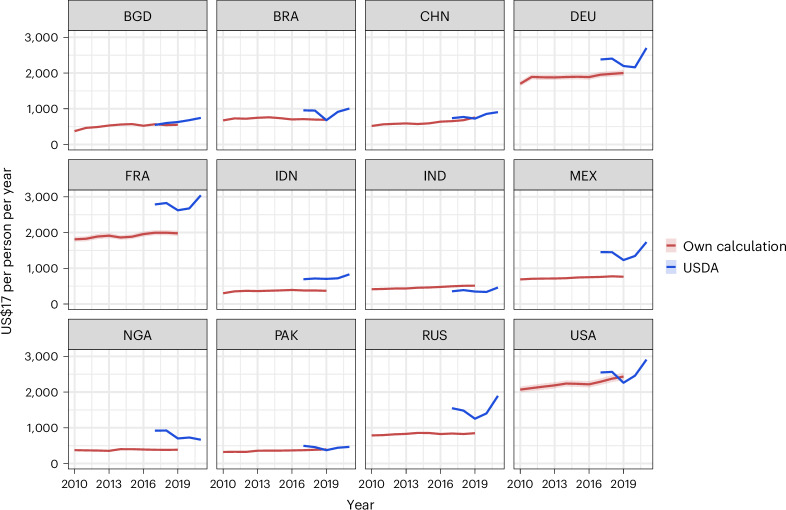
FAH expenditures per person. Per-capita FAH expenditures in US$17 for the 12 most populous countries with data from both this study (red line) and the USDA International Consumer and Food Industry Trends^6^ (blue line). Red line regression best fit applied to FAOSTAT prices. Red shaded region 95% CI. DEU, Germany; FRA, France; MEX, Mexico.

By combining producer and consumer price information with consumption data from FAOSTAT, we are able to calculate consumer expenditures across food groups, and for both FAH and FAFH. In Fig. 3, we show the FAH expenditures calculated through this method, alongside FAH expenditures published by the USDA^6^, for a selection of the most populous countries (full comparison in Supplementary Fig. 2). Despite some discrepancies, our model estimates lead to results similar to the top-down reporting collected by the USDA. Furthermore, we are able to represent country-level differences, with lower-income countries such as India and Uganda exhibiting much lower food expenditures, compared with the United States or European countries.

**Fig. 4:**
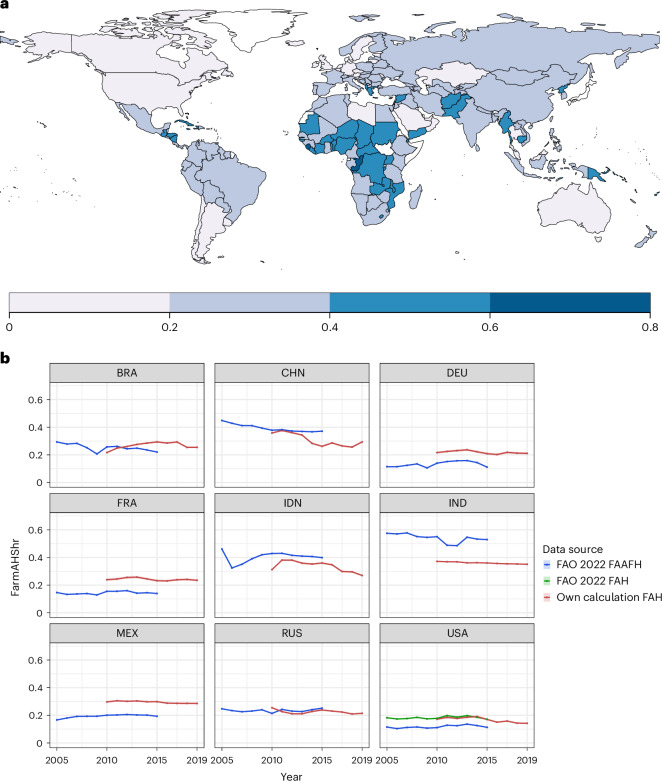
Farm share of the food dollar indicators. **a**, Farm share of the food dollar 2019. Model estimation based on all (food-at-home (FAH) and food-away-from-home (FAFH)) expenditures in 2019. **b**, Farm share of food expenditures, based on food-at-home expenditures (FarmAHshr). On the basis of FAH expenditures (red, shading 95% CI), with comparison data from FAOSTAT^24^, based on the farm shares of FAFH expenditures minus ‘accommodation and food services’ shares (blue), and FAH expenditures (green, only available for the United States). Most populous countries for which FAOSTAT data are available.

Similarly, and because expenditures calculated with our method are disaggregated by product and FAH/FAFH expenditures, we can calculate the farm share of the food dollar for all 180 countries represented in the FAOSTAT producer price data. We note strong regional differences as well (Fig. 4a), ranging from greater than 70% in sub-Saharan Africa to the United States at 13%.

Here we are also able to represent the change of the farm share for the 2010–2019 historical period, analogous to similar calculations based on input–output (I/O) data, reported in FAOSTAT^3,24^ (Fig. 4b). We note that the data shown is not perfectly comparable: I/O accounts are often limited to the ‘food and accommodation away from home’ (FAAFH) food system, for which we take the relative farm share without the ‘accommodation and food service activities’ share (blue line, Fig. 4b), to emulate the value shares that enter the FAH FVC (full comparison with all countries with data available in Supplementary Fig. 3). Only the United States has a time series of FAH as well as FAFH farm shares (green line, Fig. 4b), for which we provide further comparison in Supplementary Fig. 4. We are also able to estimate farm shares by food product. For example, farm shares in 2019 for high-income countries (World Bank definition) for ‘bread and cereals’ are on average 5% (4, 6) and for ‘eggs’ 40% (38, 41), while for low-income countries, farm shares of ‘bread and cereals’ are on average 42% (40, 43), and for eggs 88% (87, 89%) (Supplementary Fig. 5).

In our results, we observe a generally flat or decreasing trend in marketing margins for most countries, congruent with historical patterns, although our values also exhibit some rising farm shares owing to fluctuations in producer prices (that is, in Brazil; Fig. 4b). As the marketing margin rises with income, this is indicative of producer prices rising even faster than the marketing margin. Our calculation also shows year-to-year variability, stemming also from fluctuations in producer price. We note that the farm shares calculated in our approach are often higher than those calculated via the I/O approach; this difference is expanded on in ‘Discussion’.

**Fig. 5:**
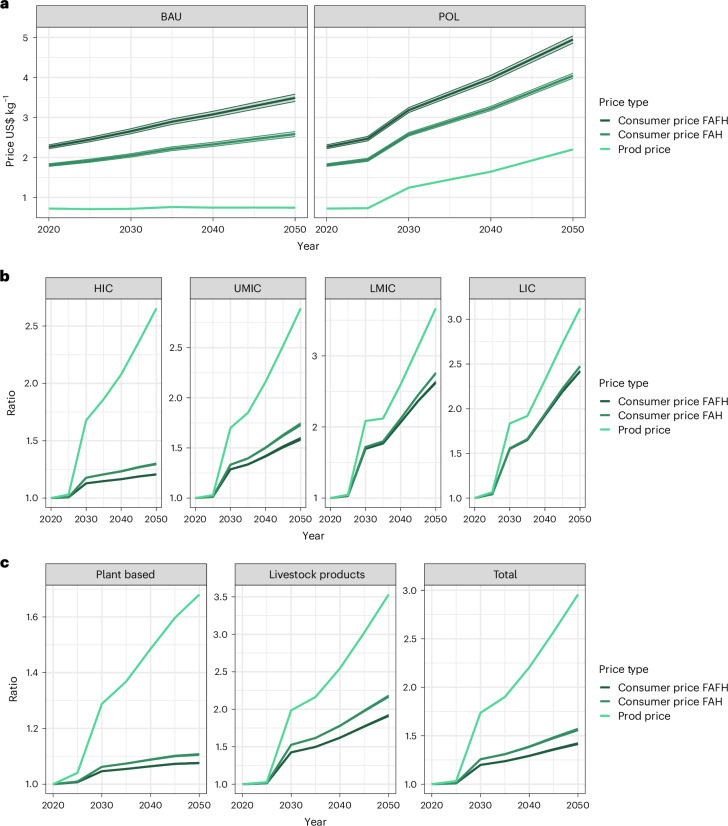
Food prices under baseline (BAU) and climate mitigation (POL) scenarios. **a**, Global average food prices: BAU and POL scenarios. Average food prices in US$ per kg for BAU and climate mitigation policy (POL) scenarios. **b**, POL-to-BAU price ratio by Income Group. Relative price changes as the ratio of POL-to-BAU prices, differentiated across aggregated income groups. Income groups based on World Bank Income Regions (high, upper-middle, lower-middle, lower income countries). **c**, POL-to-BAU price ratio by product. Relative price changes as the ratio of POL-to-BAU prices, differentiated across aggregated food groups. All prices aggregated by consumption across products and countries; shading represents 95% CI of regression results propagated.

By applying the marketing margin-to-income relationship to producer prices estimated by the MAgPIE model, we project the evolution of consumer prices into the future (Fig. 5a). In simulating an ambitious land-based GHG mitigation policy by pricing GHG emissions from farming activities, we assume a rise in producer food prices but not in the marketing margin—as marketing margin costs are assumed to be separate from land-based emissions and their associated mitigation costs. As such, while producer prices rise markedly under land-based mitigation policies, FAH and FAFH consumer prices rise relatively less, demonstrating how consumer prices are buffered by the marketing margin: added value in the FVC reduces the food price impact compared with the producer price increase. Under the POL scenario with stringent mitigation policies and high GHG prices, average global prices in 2050 in producer terms see an increase to 3.04 times 2020 values, while consumer prices only increase by factors of 2.1 and 2.2 (FAH and FAFH). The marketing margin also indicates how consumer food prices will rise in the future, in a BAU scenario. Under BAU, average food prices in 2050 in producer price terms will stay relatively constant at 1.03 times 2020 values, while consumer prices rise to 1.42 and 1.54 times 2020 values for FAH and FAFH, respectively, given future socio-economic development.

There is an important difference between how land-based mitigation policies affect producer and consumer prices in different regions of the world (Fig. 5b). As the farm share is much higher in lower-income regions, any increase in producer prices will lead to stronger relative increases in overall food prices; in other words, the producer price impacts still lead to strong effects on food consumers. In low-income regions, consumer prices by 2050 under a climate change mitigation scenario are nevertheless 2.42 and 2.48 times higher than under BAU for FAH and FAFH, slightly less than the 3.1 times higher value estimated for producer prices. In high-income regions however, producer prices are more than double BAU prices, at 2.65 times by 2050 under the POL scenario, but consumers only see an increase by factors of 1.29 and 1.21 (FAH and FAFH) between BAU and POL.

**Fig. 6:**
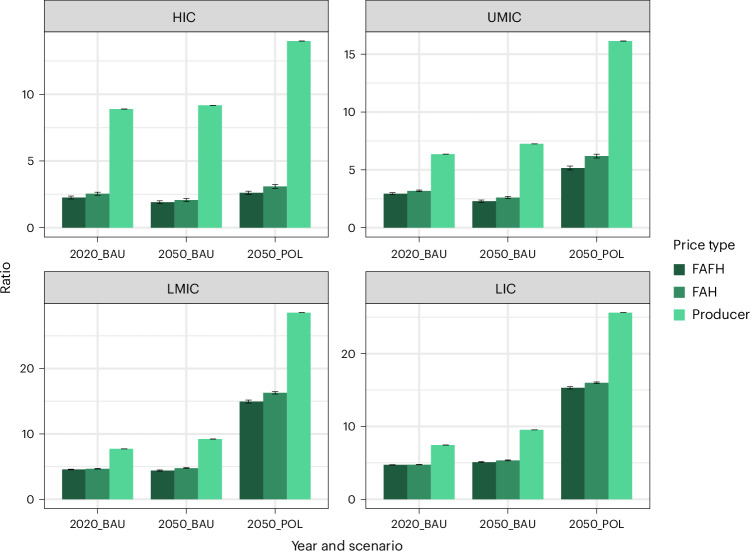
Price ratio of livestock- to plant-based products. Aggregated based on tonnes DM consumed for 2020 (2020_BAU) and 2050 BAU (2050_BAU) and POL (2050_POL) scenarios, across high-income (HIC), upper-middle income (UMIC), lower-middle income (LMIC) and low-income (LIC) countries, as defined by the World Bank. Bars portray the price of animal-based products divided by the price of non-animal-based products, that is, a relative difference between the two prices. The three shaded bars indicate this relative difference across the three types of prices calculated in this study through MAgPIE in conjunction with the marketing margin, with the price ratio for FAFH products the darkest shading, FAH in medium shading and the producer price ratio in lightest shading. Note how the ratio is much higher when (1) considering lower-income countries and (2) under a policy scenario, considering producer prices as opposed to either of the two consumer prices. Error bars represent 95% CI of regression results propagated, *n* = 249 countries distributed across income groups.

In comparing the relative changes between food products (Fig. 5c), now aggregated by plant- and animal-based products, we note the same effects, and in particular the strong increase in prices of animal-based products stemming from the higher-emissions factors of animal husbandry. Again, consumer prices of animal products are less affected than producer prices, with the producer price of animal-based products more than tripling by 2050, while consumer prices only double. A different consumer price change of livestock products has implications for the demand-side income effect of climate policy on consumption of livestock products, while the relative difference between livestock- and plant-based foods is important for the substitution effect. As such, we show in Fig. 6 how the ratio between livestock and plant-based products evolves under a stringent mitigation policy, considering consumer prices. In high-income countries, the ratio of livestock to plant products’ producer prices are nearly double in terms of the POL-to-BAU scenario (14.0 versus 8.89) by 2050. In other words, livestock products in terms of producer prices become more than 14 times more expensive than plant-based products under a policy scenario, and are 8.9 times more expensive otherwise. However, in terms of FAH and FAFH prices, the Livestock to Plant price ratio only sees ratios between POL and BAU of 3.1 versus 2.44 (FAH), and 2.61 versus 2.16 (FAFH). This pattern can be observed for all world regions (Fig. 6).

## Discussion

By combining a statistical and process-based modelling approach, we have estimated the evolution of food prices over time, across food products and countries, including both producer prices as well as FAH and FAFH prices. These results provide extensive geographic and product coverage, building on recent work based on more aggregate data^3,24^.

Across the various food products, the result that bread and cereals as well as meat products see higher marketing margins at higher per-capita income is a dynamic highly indicative of the ‘nutrition transition’, as consumers begin purchasing foods at retailers with higher levels of standardization, processing and marketing^2^. The marketing margin described in this study is quite broad: it captures how consumers spend more on the non-agricultural share of food as economies develop, but owing to the already-aggregated nature of the data, we cannot split between the various components the margin includes, beyond the FAFH component. However, the relative difference in how the margins change between products is indicative of how these products are treated in the agri-food chain. Notably, the higher margins for ‘bread and cereals’ and meat products likely stem from the higher amounts of processing that these products undergo, as highly advanced food processing technologies allow for the transformation of these commodities into more convenient, less perishable versions. In the case of cereal products, basic grains can become very highly processed, becoming bakery products, breakfast cereals and pasta, for example, and meats via curing, drying, canning, ready-to-eat preparations and so on. As is well documented, this also often decreases the nutritional value of these ingredients and leads to a ‘paradoxical’ decline in health outcomes even as incomes rise^25^. As the data used in this paper classifies foods based on their main ingredient, inaccuracies can also stem from high processing, in the case of processed meat products or ready-to-eat meals, for example, as in the ICP dataset these are based on their main ingredient. (Note that the ‘processed’ category does not include such goods, and only contains food products that are based primarily on sugars and oils; Methods.) This can lead to some inaccuracy across food groups, with upward bias of the margin for the main ingredient and downward bias for the secondary ingredients that are still part of such products. However, in comparing our price per unit results, we see that these remain comparable to observations such as those of the USDA^26^. In terms of uncertainty, mutton products see higher uncertainty owing to the lower number of observations. ‘Milk products’ see high uncertainty and lower marketing margin values, possibly as milk is both consumed with hardly any processing, but also transformed into cheese and other dairy products. Furthermore, processing into products such as cream and butter often already happens at the farm level, owing to the existence of producer prices for these products in the FAOSTAT database, which is accounted for in our calculation. Eggs see high uncertainty but are indeed also mixed into ready-to-eat preparations as well as heavily consumed unprocessed. Fruits and vegetables see only a moderate marketing margin and indeed are often purchased whole and fresh, or only lightly processed through freezing and preservation.

FAFH is more expensive than FAH, and consumption of FAFH also increases as incomes rise, as expected. While these combined dynamics lead to higher food expenditures in higher-income countries, overall incomes are even higher, leading to decreasing farm shares of the food dollar. Of course, while the value of the farm share decreases, on-farm activities are an integral aspect of food production, and on-farm production will retain its primacy in terms of physical production, as well as in dynamics such as employment in lower-income countries, and land-use and other environmental impact footprints^27^.

As shown in Fig. 4b, our estimates of the farm share of the food dollar can deviate from calculations based on I/O tables^24^ (Supplementary Fig. 3). This can stem from various uncertainties in both methods, with some further uncertainties for this study discussed at the end of this section. We also note that for the United States, our value is congruent with the farm share for total expenditures, that is, FAH with FAAFH (Supplementary Fig. 4, as opposed to the FAH values compared in Supplementary Fig. 3, owing to data availability). Product-based farm share values for the United States are also available^28^, although not directly comparable for all products as these data report unprocessed ingredients and not their processed final consumer products, that is, ‘beef’, with very high farm shares (of 52.5%, for beef), as opposed to beef and all processed beef products. However, for a more comparable product, we can look to eggs, where our value of 31.1% (29.8–32.6%) is in accordance, though still much lower than the 58% reported in 2013^28^. Here again, slightly more processing in the ICP database may be the source of this discrepancy, as the overall farm share of 17% (ref. ^28^) is very much in line with that in this work for the United States, of 18.8 (18.6–19.1%).

Furthermore, other empirical studies show higher farm shares in line with our estimates, for example, for the United Kingdom at 36–41% for 2015–2017^29^, France at around 20% in 2011^30^ and Germany with 23% in 2014^31^. Finally, our approach allows for a general overview of global farm shares by food group, owing to our product-differentiated marketing margins (Supplementary Fig. 5), showing clearly how producers in different FVCs receive very different shares of the consumer dollar, also highlighted in specific country-based studies^28,30–34^.

With regard to future projections, we note that producer prices do not see stark increases in the future under a baseline scenario, as the model mimics real-world investments in more efficient production methods^35^. This is congruent with historic trends, where technological changes have led to stabilization and even decline of agricultural commodity prices over the past decades^36^. However, we observe that as economic development takes place, an increasing share of consumer expenditures is indeed directed towards food-based services as opposed to raw commodities. Interestingly, in the policy case, we observe that the cost of agricultural production given the very high carbon prices (more than US$500 per ton CO_2_–C in 2050, and US$700 per ton CO_2_ by 2100) also leads to the farm share increasing again, but only at the beginning of implementation. The farm share plateaus once the carbon tax reaches around US$200 per ton CO_2_ (between 2030 and 2040, differentiated across countries’ economic development), and again begins to fall, but later in the century as markup and consumption dynamics again take over. We present a plot of this dynamic (CO_2_ price plotted against farm share in the policy case) in Supplementary Fig. 6.

We acknowledge that strong market inefficiencies and market power exist across the FVC^37^. In terms of agricultural producers and the associated input markets, MAgPIE assumes perfect competition, with producer prices given efficient markets. However, the marketing margins are statistically computed based on historical empirical data, thus implicitly including existing vertical price transmission effects. As such, market dynamics are implicitly included in the post-farmgate downstream processes where market power effects may be most prevalent (but also more complex)^37,38^. By extending the regression results into the future, we assume that current market structures are extended into the future. Furthermore, in the case of emissions pricing in the land-use sector, the fact that MAgPIE does not account for market concentration and the distribution of price increases among actors in the supply chain up to the farmgate implies complete pass-through of producer cost increases to consumers. This price transmission follows a theoretical model of atomistic markets, while in reality market dynamics, including oligopolistic structures, as well as psychological pricing strategies result in price transmissions that are much more complex^37^.

In terms of the potential food security impact of climate mitigation policies, we show here the isolated effect on food consumers of a high emissions tax on the AFOLU sector, that is, land and agricultural production. This can be seen as isolating the ‘additional burden’ of applying GHG taxation (including non-CO_2_ taxation) to the land sector. This provides insight into the degree of burden that food consumers would face, especially if all tax burdens are passed on to consumers. This is especially important, as only 18% of food system emissions come from non-AFOLU activity, such as production of inputs, processing of agricultural products, transport, packaging and retail, while 72% of food system emissions do stem from (currently unpriced) AFOLU emissions^39^. Under an economy-wide climate mitigation policy, costs of energy-intensive supply chain components such as cold storage and transport in the food system would also increase, affecting the marketing margin, whereby the margin would also increase between POL and BAU. However, this portion of food system emissions is less than a quarter of AFOLU emissions, and GHG intensities of these sectors would also decline over time given policy implementation. Given the paucity of data on value chain shares and emissions intensities in the food system, and how these may change with the decarbonization of the energy system, we present a land-based AFOLU mitigation scenario along the lines of previous studies. A disaggregated study of how multi-sectoral climate change mitigation policies will impact the various parts of the FVC is an important topic for future research.

The disaggregated nature of our estimates allows for a nuanced analysis of how climate mitigation policies may affect food consumers across countries and food sectors. Indeed, these considerations would apply for any policy or outside intervention that may affect agricultural production costs and prices, such as changing fertilizer, energy, irrigation or labour costs^40^. In lower-income regions where food security considerations are most salient, value added in FVCs is very low, and FAH prices in low-income countries rise almost as much as the producer price in the POL scenario (Fig. 5c). This highlights the equity and welfare considerations that will be necessary for a fair implementation of global emissions prices, as consumers in higher-income countries will be relatively less affected than those in lower-income countries, where both a larger share of household expenditures is spent on food, and consumer food prices are more strongly tied to producer prices. Even in higher-income countries, changing producer prices may lead to retailers increasing pass-through to consumers beyond the costs they face, as indicated in recent discussions of ‘greedflation’^41^. This dynamic could not be captured in this study, as we assume a price-taking case where all costs are passed through to consumers. As such, within- and across-country redistribution of the tax rents from emissions pricing, or regionally differentiated emissions prices, would need to consider these components when seeking to defray the costs of mitigation policies on the most vulnerable^42^.

There will be demand-side effects on consumption that were not accounted for in our current modelling framework, as food demand in the MAgPIE model is inelastic. Increasing food prices generally leads to both income and substitution effects. As livestock products embody higher GHG emissions, the price of these will increase, both absolutely and relative to plant-based products. The absolute increase would lead consumers to consume less livestock products based on the own-price elasticity, while the relative increase would lead to decreased livestock consumption in favour of plant-based products. Both effects would lead to demand-side mitigation; however, here we show that this effect would also be less when considering consumer prices (Figs. 5b and 6).

In terms of the marketing margin itself, a more robust modelling approach would capture agents’ preferences in terms of the various components that make up the margin, and analyse how actors along the FVC as well as consumers respond to income and price changes. In this study and with regard to data limitations, we can only capture one component of the FVC explicitly, that is, food consumed away from home, which the best-fit regression found a value-based margin to fit best. For the rest of the margin, as mentioned, we applied a difference-based marketing margin as many components of the marketing margin appear to be volume-based as opposed to proportional to value, based on the data. In other words, value added is applied to products based on mass, as opposed to monetary value. The alternative, value-based approach led to data that did not show any discernible pattern nor relation with GDP (Supplementary Fig. 7), leading us to conclude that the bulk of the margin in this case does stem from volume-based processes. With appropriate data, to better represent real-world dynamics, further volumetric and money metric components of the value added could also be separated out and modelled independently.

Some uncertainties also emerge with the modelling approach used here. We assume that the product categories are homogeneous in nature across countries, which is not necessarily true and can bias the estimations. Furthermore, our calculation of the marketing margins leads to a few negative values—where the consumer price is cheaper than the producer price. This is unintuitive in reality and may stem from several sources: (1) consumer foods may be subsidized to reduce prices below production costs; (2) products included in the FAOSTAT price survey may be of higher quality than what people more typically purchase, especially from more informal markets, leading to increased producer prices; (3) inconsistent data reporting and classification between the ICP and FAOSTAT, again stemming from the fact that certain cheaper ingredients may be included in the more processed foods in the ICP dataset; (4) despite these uncertainties, we keep the negative values to avoid biasing the overall distribution of the data or the regression outcomes, and to present our results in the most transparent manner possible. The hierarchical nature of the statistical model applied also diminishes the importance of these points (although does not solve the issue): ‘shrinkage’ effects, which refer to the process of pulling extreme values towards the overall mean, allow for model predictions to be informed by the entire dataset, reducing the importance of any individual data points within and between grouping categories (that is, food group or location of consumption)^43^.

The calculation of marketing margins in this study highlights the importance of the added-value components in the formation of food prices, especially in the future as countries increasingly shift towards more complex food systems. To assess the robustness of the overall dynamic presented in this paper, we conduct a sensitivity analysis along all five shared socio-economic pathways (SSPs)^44^ for both BAU and POL scenarios, where different future scenarios (sustainable and internationally co-operative, or otherwise) are used to drive the model results (see sensitivity analysis in Methods and Supplementary Table 2 and Supplementary Figs. 8 and 9). From this exercise, we conclude that similar dynamics, that is, differing impacts on consumers between high-income and lower-income countries, also remain, even in alternative futures.

Our analysis thus provides a first glimpse at how climate mitigation policies may impact food security differently when including consumer prices, and is especially relevant for model-based approaches where price elasticities are based on consumer expenditures^5^. We demonstrate that incorporating consumer prices explicitly is essential when considering questions of climate change mitigation, both for the food price impacts as well as demand-side changes. However, one reason for this gap is the lack of data available for FVC components^10^. Transparent and easily accessible data that covers a broad span of temporal, spatial, and processing and marketing dimensions is rare for FVCs^3,11^. While this study uses a model-based approach to infer some of this data, more empirical research will also be fundamental in achieving a better understanding of the food system as a whole.

## Methods

### Calculation and regression of marketing margins

We calculate the marketing margin based on existing data of consumer food expenditures^45^, consumption volumes^46^ and producer prices^22^. We combine consumer food expenditures stemming from the World Bank’s ICP dataset^45^, which tracks consumer expenditures by product, with the UN Food and Agriculture database’s (FAOSTAT) producer price^22^ and food balance sheet dataset^46^. This allows for a comparison between consumer prices and producer prices, with the difference between the two being the marketing margin.

Owing to differences in data reporting, several steps of pre-processing and aggregation are required. On the ICP side, we use the consumer expenditures (which does not include consumption volumes) at 1 aggregate product levels: ‘bread and cereals’, ‘rice’, ‘beef’, ‘poultry’, ‘pork’, ‘lamb and mutton’, ‘fruit’, ‘vegetables’, ‘oils’, ‘sugar, jam, honey, chocolate and confectionery’, ‘milk and milk products’, ‘eggs’ and ‘food products n.e.c. (not elsewhere classified)’. The ‘food products n.e.c.’ category is distributed to the rest of the products, based proportionally on each country’s expenditure shares. As the ICP database also reports expenditures on FAFH as a separate ‘food group’, we also allocate this value to all product categories proportionally after distributing the ‘food products n.e.c.’, following the naive assumption that the shares of foods consumed remain the same for FAH and FAFH. Finally, the ‘oils’ and ‘sugar, jam, honey, chocolate and confectionery’ categories are summed to a ‘processed’ category to facilitate comparison. ICP data is collected once every 6 years; here we use the most recent data from 2017 and 2011, for 138 countries and territories. Expenditures are converted into constant 2017 USD MER. To derive prices from the expenditures that are then comparable to FAOSTAT, we map the ICP products to their raw commodity equivalents and divide them by volumes consumed, from the FAOSTAT food balance sheets, in terms of raw commodity. In terms of consumption of the ‘processed’ category, which refers to sugars and oils, we use the weighted demand of primary products that go into sugar and oil processing (sugar cane and sugar beet, and oil crops, respectively), based on national-level consumption and conversion factors from FAOSTAT.

We use FAOSTAT producer prices and food consumption to compare against the prices derived from the ICP. FAOSTAT reports producer prices and consumption at the level of 212 product categories; we map these to the same product categories as used for the ICP and aggregate them based on consumption from the balance sheets, allowing for comparison with the ICP prices, now based on the same volumes. Note that we exclude non-food products as well as beverages such as teas and alcohol from both ICP and FAOSTAT datasets.

The difference between the ICP and FAOSTAT expenditure is then divided by the consumption in tons of primary product for each product category to derive a marketing margin per ton of primary product. As the marketing margin is now calculated per ton of primary product, any change from primary to final product in terms of loss or waste is implicit in the marketing margin value.

### Modelling framework

We apply a hierarchical regression using a Bayesian approach, modelling consumer price marketing margins as the outcome of a nonlinear function on the natural logarithm of per-capita income, with varying effects for product group as well as for location of consumption (FAH and FAFH). As we have two separate years represented in the data, we specify a year dummy variable to take time effects independent of the income effect into account. Given the paucity of years compared with country data points, we omit taking any time-independent country effects into the regression. As the distribution of marketing margins sees outliers and wider tails, we specify a student-*t* distribution with 3 degrees of freedom, which provides a better match to the data. Owing to the non-normality of country incomes, we also take the natural logarithm of per-capita income. As the data exhibits nonlinear dynamics for most products, as well as some negative values in the marketing margins, we use the following exponential specification of the regression equation. This functional form (equation (1)) allows us to first estimate the regression parameters and then transform the parameters to the log–log scale by taking the log of both sides of the regression equation, and avoiding any potentially biased conversions of the negative marketing margin values^47^. ln(*b*) is thus interpretable as an income elasticity.

1 $${{\boldsymbol{Y}}}_{k,c} \sim \mathrm{Student},\left(3,{a}_{k,c}\times {{b}_{k}}^{{{\mathbf{lnGD}}}{{{\mathbf{P}}}}_{{{\mathbf{pc}}}}},\sigma\right)+{\mathrm{year}}$$

2 $${a}_{k,c} \sim N(\,\mu ,\sigma )$$

3 $${b}_{k} \sim N(\,\mu ,\sigma )$$

where *Y* is the markup values, *k* is the food groups and *c* is the location of consumption (FAH/FAFH).

Priors for unknown parameters *a* and *b* as well as their standard deviations within the two grouping variables were regularizing to aid convergence and only slightly informative (see Supplementary Note 1 for detailed prior specifications). Data points are weighted by country population, to draw predictions towards more populous (important) countries. Parameters were estimated using Monte Carlo Markov chain sampling, with 4 chains of 4,000 draws (2,000 warmup discarded). Chains were checked for convergence both visually and with Rhat values being equal to 1; model and prior specification were also confirmed with prior and posterior predictive checks (Supplementary Fig. 10). All regressions were undertaken using the *brms* R package (version 2.19), an interface to the STAN Bayesian inference engine^43,48^.

Because we derive marketing margin coefficients and estimates for both FAH and FAFH, we split FAOSTAT consumption into FAH and FAFH as well. On the basis of a literature review, we are able to identify the share of FAFH consumed as the share of total calories consumed, for only 14 countries. These are regressed based on a simple linear regression on per-capita income (Supplementary Fig. 11). The regression-derived estimate is applied to all countries for which actual data does not exist, while the country value is kept for those that do exist. The regression estimate is also used for future projections as country incomes change; countries for which data exists move up the slope from their initial value. In the absence of sufficient cross-country data, the shares of calories from various food groups are assumed to be the same across FAH and FAFH.

We estimate the farm share by applying producer, FAH and FAFH prices to FAH and FAFH consumption levels in each country based on FAOSTAT food balances, and dividing by the total expenditures. All analysis is conducted in real prices with 2005 as base year using the R package GDPuc^49^.

The marketing margin-income relationship is incorporated into the MAgPIE modelling framework (Model of Agricultural Production and its Impact on the Environment)^21^, and the product-specific margins are applied to the model’s endogenous agricultural (producer) prices.

MAgPIE is an open-source PE global land-use modelling framework^21,50^. It integrates biophysical information, such as potential crop yields, water availability and carbon stocks, along with socio-economic data such as income, population, and input and commodity prices. The model must meet global food, feed and material demand^4^ with various decisions to be made on the basis of cost minimization. We apply the MAgPIE model for two scenarios, the BAU scenario as well as the one with emissions taxation policies POL. In both scenarios, the primary external drivers of the model are future population and GDP trajectories, both based on the SSP2 scenario, which is part of the SSPs set characterizing future challenges to climate change mitigation and adaptation^44^. For a detailed description of how other model components such as food consumption, technological change, protected areas or international change under the SSP2 scenario, see Supplementary Table 2. The SSP2 narrative translates into moderate levels of GDP and population growth into the future. Final food and feed demand, categorized into 19 different crop groups and 5 livestock groups, is derived based on a food demand module, and the model then finds a plausible future pathway based on minimizing global costs while subject to various constraints, as stated. These costs include production, labour and capital costs of agricultural activities, as well as costs for inputs, trade, land conversion and so on. Producer prices in MAgPIE are derived as the shadow price of demand, that is, the marginal cost of supplying one unit further in terms of production. The producer prices reported in this study reflect the possibility of international trade as well, such that the marginal cost is the lesser of producing one more unit domestically or importing one more unit produced in another region.

The difference between BAU and POL in this study is that, starting in 2025, an additional cost—a tax on GHG emissions produced through agricultural activities and through land-use change—is imposed, along with increased demand for bioenergy crops. This policy scenario is implemented with exogenous GHG price and bioenergy demand trajectories that allow for the reaching of the 1.5 degree warming target. These trajectories were previously determined endogenously by coupling MAgPIE with REMIND, an energy-sector/macro-economy model^51^. Demand for first- and second-generation bioenergy is also determined via the MAgPIE-REMIND coupling, which exchanges information on land scarcity, and bioenergy demand between the two models, allowing for the calculation of optimal price trajectories^51^. Carbon prices are introduced at different levels reflecting the regions’ different abilities to pay, with carbon prices in higher-income regions increasing linearly and lower prices in lower-income regions, reaching a uniform price by 2050.

This price on emissions leads to differing model behaviour between the two scenarios. In the case of carbon dioxide emissions, the emissions tax makes it costlier to convert forest or other natural land into cropland, thereby disincentivizing land conversion and favouring investments in productivity instead. In the case of nitrogen and methane emissions, which stem from activities such as fertilizer applications, enteric fermentation of ruminants, animal waste treatment and rice cultivation, the model can invest in emissions abatement based on long-term marginal abatement cost curves^52^. This leads to an optimal level of emissions production based on the level of the emissions tax, while costs of production and thereby producer prices will increase owing to the increased investment required.

Model runs were performed with MAgPIE version 4.7.2, available here: https://github.com/magpiemodel/magpie/releases/tag/v4.7.2.

### Uncertainty and sensitivity analysis

To assess the robustness of the results, we portray the uncertainty and sensitivity in the results via two approaches, statistical and scenario-based. The statistical uncertainty is based on the propagation of the 95% CI of the regression through the calculated results, allowing for an understanding and visualization of the band where projections are 95% most likely to lie, based on the uncertainty in the regression data.

Furthermore, a broader scenario-based sensitivity analysis is conducted for the MAgPIE-calculated producer prices, which components such as trade margins and tariffs that may change in the future. To do this, we run the MAgPIE model for BAU and POL scenarios along the full set of five SSP scenarios. These scenarios describe various possible futures, ranging from a more sustainable future with international co-operation to an unsustainable future with isolated and competitive nation-states. Full descriptions for how these scenarios enter and drive MAgPIE are also found in Supplementary Table 2, along with results in Supplementary Figs. 8 and 9.

### Reporting summary

Further information on research design is available in the Nature Portfolio Reporting Summary linked to this article.

## Supplementary information

Supplementary Information Supplementary Tables 1 and 2, Note 1 and Figs. 1–11.

Reporting Summary

## Data Availability

Data on producer prices are publicly available online here: https://www.fao.org/faostat/en/#data/PP (ref. ^23^). The consumer expenditure data is available at coarser aggregation here: https://databank.worldbank.org/source/icp-2017. This study had access to more disaggregate data upon which we are under confidentiality agreement with the ICP. The data is available from the ICP upon request. All further datasets and mappings used are archived within the custom R package: 10.5281/zenodo.12926687 (ref. ^53^). Initial data processing and analysis, that is, calculation of marketing margins, were conducted via a custom R package, archived through GitHub here: 10.5281/zenodo.12926687 (ref. ^53^). Output analysis and replication scripts, including MAgPIE output folders, are archived at 10.5281/zenodo.12927368 (ref. ^54^). All publicly available datasets are archived within the R package.

## Code availability

MAgPIE is an open-source model available at https://github.com/magpiemodel/magpie. The model documentation for the exact version of MAgPIE used in this study (v.4.7.2) can be found at https://github.com/magpiemodel/magpie/releases/tag/v4.7.2. All other code is publicly archived at 10.5281/zenodo.12926687 and 10.5281/zenodo.12927368.
