# Supplementary material for: Future food prices will become less sensitive to agricultural market prices and mitigation costs
Source: Nat Food. 2025 Jan 3;6(1):85–96. doi: 10.1038/s43016-024-01099-3 (PMC11772223; doi:10.1038/s43016-024-01099-3)
Supplement: Supplementary file 1 — Supplementary Tables 1 and 2, Note 1 and Figs. 1–11. [file 43016_2024_1099_MOESM1_ESM.pdf]

nature food

Supplementary information https://doi.org/10.1038/s43016-024-01099-3

Future food prices will become less sensitive to agricultural market prices and mitigation costs

In the format provided by the authors and unedited

Supplementary Information

Table of Contents:

Supplementary Table 1-2 2

Supplementary Note 1 Prior Specification...4

Supplementary Figures 1-11 5

Supplementary Table 1. Income Elasticities. Estimate and 95% CI bounds.

	Estimate	Q2.5	Q97.5
Beef and veal	1.45	1.429	1.471
Bread and cereals	1.418	1.397	1.439
Eggs	1.326	1.303	1.348
Fruit	1.316	1.291	1.34
Lamb, mutton and goat	1.506	1.483	1.529
Milk products	1.197	1.173	1.222
Pork	1.414	1.394	1.435
Poultry	1.399	1.379	1.42
Processed	1.267	1.245	1.289
Rice	1.166	1.132	1.199
Vegetables	1.263	1.241	1.286

Supplementary Table 2. MAgPIE Driver Description. MAgPIE drivers and parametrizations important to this study, across the 5 Shared-Socioeconomic Pathway (SSP) scenarios used in sensitivity analysis.

MAgPIE drivers and parametrizations	SSP1	SSP2 (Business-As-Usual)	SSP3	SSP4	SSP5
**Population and Income: Exogenous**	Low population growth, high income growth rates	Moderate population and income growth rates	High population growth, low income growth rates	Moderate population growth, low income growth rates	Low population growth, high income growth rates
**Diets: Driven by per capita income and the demography of the world population**	Healthy and low-animal product diets, reduced food waste	Along historic trajectories of animal-product consumption and food waste	Unhealthy and high animal-product diets, high shares of food waste	Along historic trajectories of animal-product consumption and food waste	Unhealthy and high meat consumption diets, high shares of food waste
**Management and Technonological progress: Endogenous irrigated versus reainfed crop production management. Endogenous intensification of inputs in the production. Different levels of R&D and costs**	low costs	Moderate costs	high costs	high costs	low costs
**Protected areas: Exogenous. Land protection based on the (WDPA), with different fade-in protection policies.**	High coverage	Moderate Coverage	Low coverage	Low coverage	High coverage
**Bioenergy: Exogenous, based on coupled runs made with REMIND**	High second generation bioenergy demand	Moderate second generation bioenergy demand	Moderate second generation bioenergy demand	Moderate second generation bioenergy demand	High demand
**Trade: Endogenous. Historical patterns until 2015 (FAO), after which free trade based on competitive advantage happens for scenario-specific percentage of total trade. Trade margins stay constant while trade tariffs decrease at a scenario-specific rate.**	Free trade occurs for 20% for livestock and secondary products, and 30% for all other traded commodities by 2050	Free trade occurs for 20% for livestock and secondary products, and 30% for all other traded commodities by 2050	Free trade occurs for 5% for livestock and secondary products, and 10% for all other traded commodities by 2050	brmRev1OutConsTighterHigherFilterpop1outliPopWSplit21Y7	Reaches 5% for livestock and secondary products, and 10% for all other traded commodities in 2050, until 2100

Supplementary Note 1: Prior Specification

We specify a *student-t* distribution to model the consumer price markups, due to the presence of outliers and wider tails in the distribution of the markups. The *student-t* distribution is parametrized with 3 degrees of freedom, and mean μ and standard deviation σ. The markups interact with the natural log of gdp based on a nonlinear relationship (Eqn. 1). Coefficient *a* is allowed to vary between both food groups and location of consumption, while *b* varies only between food groups. The standard deviation of these coefficients is assumed to be normally distributed, with a normal prior, while σ, the standard deviation of the residuals, is specified with a wider, student-t scaled to the data.

Priors (eqns. 2-4) were assigned and optimized through an iterative process, where prior predictive checks (sampling only from the prior and not the data) were used to scale the priors in order to facilitate model convergence. In this way, the priors were regularizing, but given the data, only slightly informative. In Supplementary Figure 7, we see the prior and posterior predictive checks (i.e. sampling from the prior only, vs sampling from the model specified on the data), showing that the prior provides a very wide range of possible outcomes distributions, while the specified model based on the data has converged to a relatively good fit of the outcome distribution.

$$\mathbf{Y}_{\boldsymbol{k,c}} \sim \boldsymbol{student}(\mathbf{3},\ \boldsymbol{a}_{\boldsymbol{k,C}} * \boldsymbol{b}_{\boldsymbol{k}}^{\mathbf{lnGDPpc}} + \boldsymbol{c}_{\boldsymbol{year}}, \boldsymbol{\sigma}) \quad \mathbf{(1)}$$

$$a_{k,c} \sim N(0.1,\ 0.05) \quad (2)$$

$$b_k \sim N(1,1) \quad (3)$$

$$c_{year} \sim N(1,1) \quad (4)$$

$$\sigma \sim student(3, 300, 800) \quad (5)$$

where

Y: $Markup\ values$

k: $Food\ Groups$

C: $Location\ of\ consumption\ (FoodAtHome/FoodAwayFromHome)$

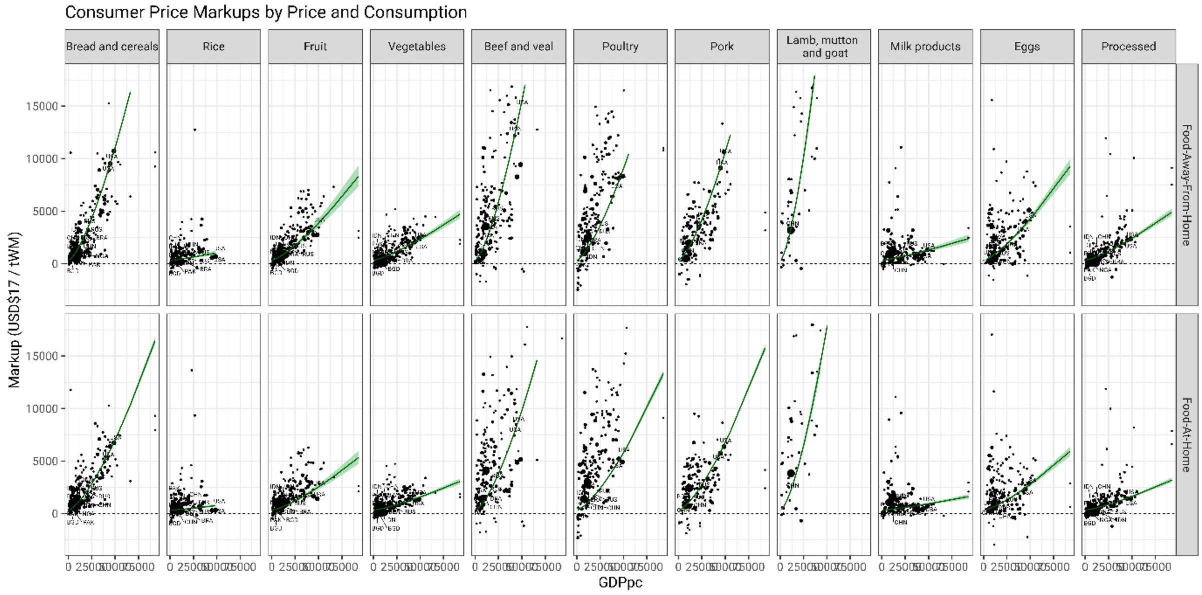

Supplementary Figure 1. Identical figure to Fig. 1 of main text, with markups and fitted model, with x-axis on unit scale as opposed to log scale, shading 95% credible interval of regression mean.

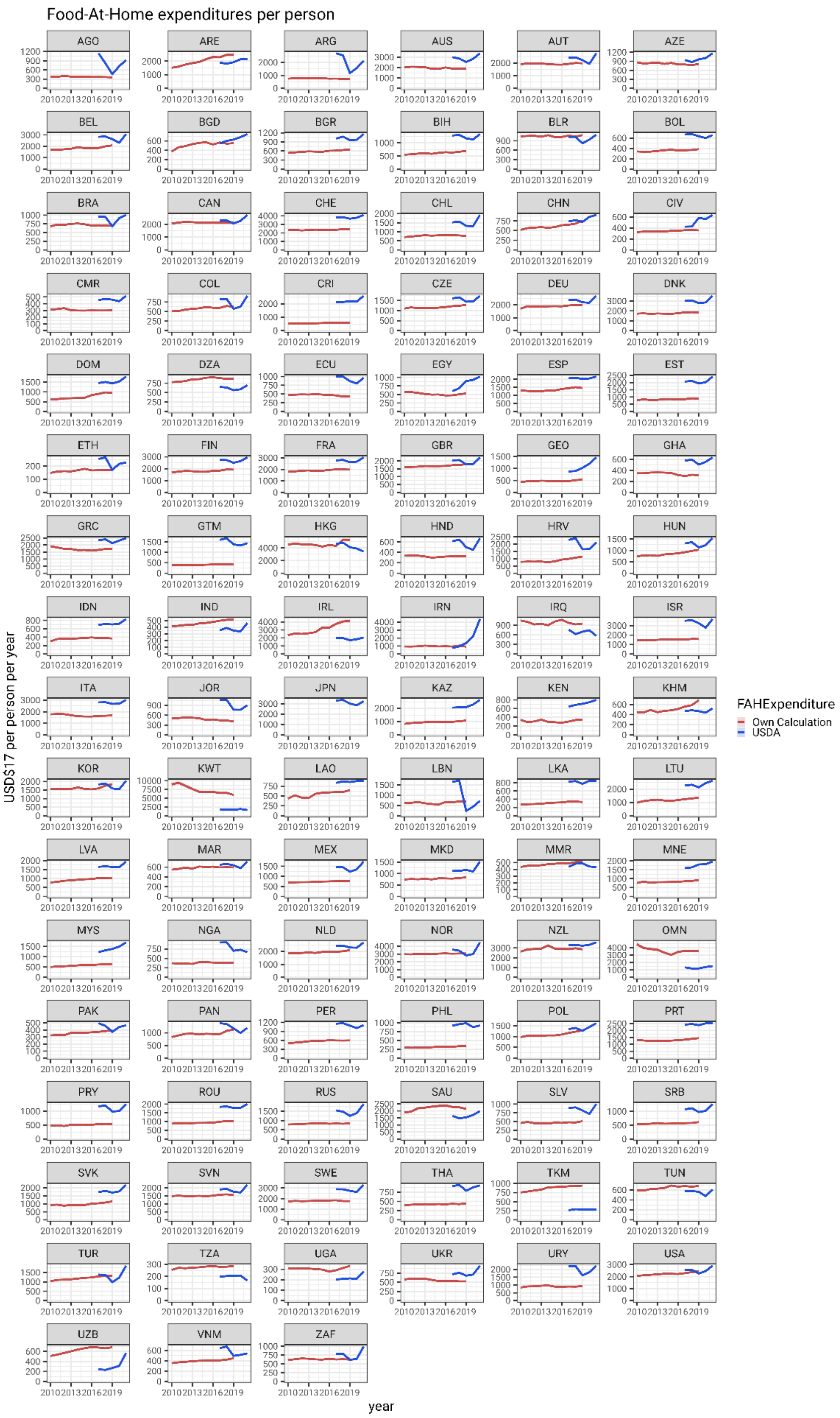

Supplementary Figure 2. Food-At-Home expenditures for all countries existing in USDA 2021 data (blue), compared to own estimates (red, shading 95% credible interval), based on regression markups and FAOSTAT producer prices.

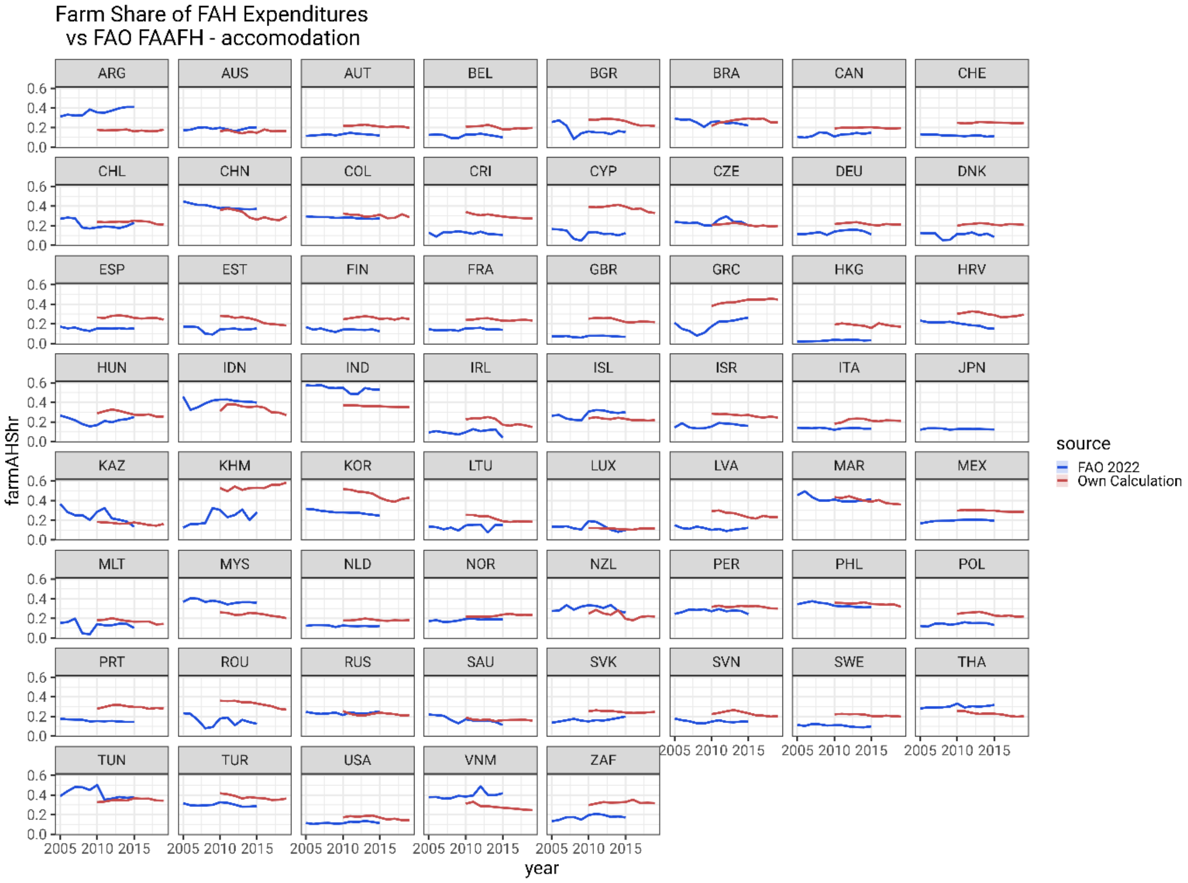

Supplementary Figure 3. Farm share of FAH Expenditures (red, shaded area 95% Credible Interval), compared to FAOSTAT (2022) farm shares (blue) based on FAAFH Accomodation and Food Services value share subtracted from total.

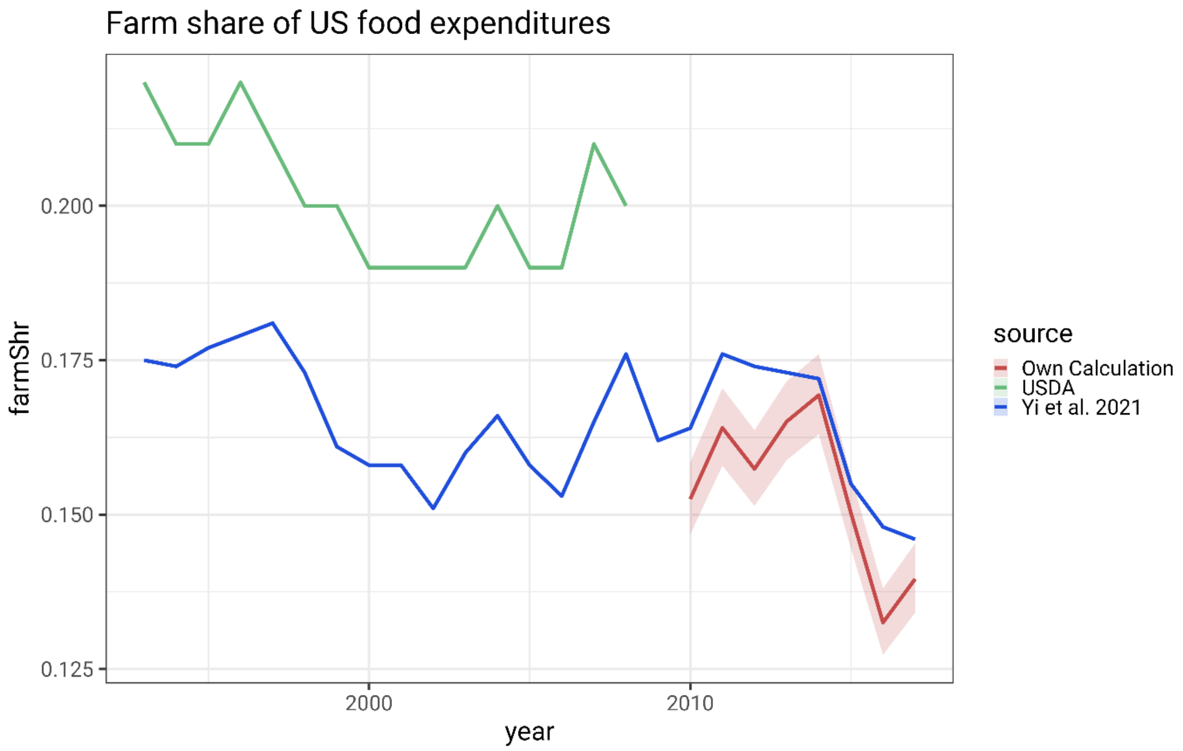

Supplementary Figure 4. Farm share of US food expenditures for total expenditures (FAH + FAFH). Datasources based on own calculation (red line model-based estimate, shading 95% credible interval) and Yi et al. (2021) which provides both

blue and green line.

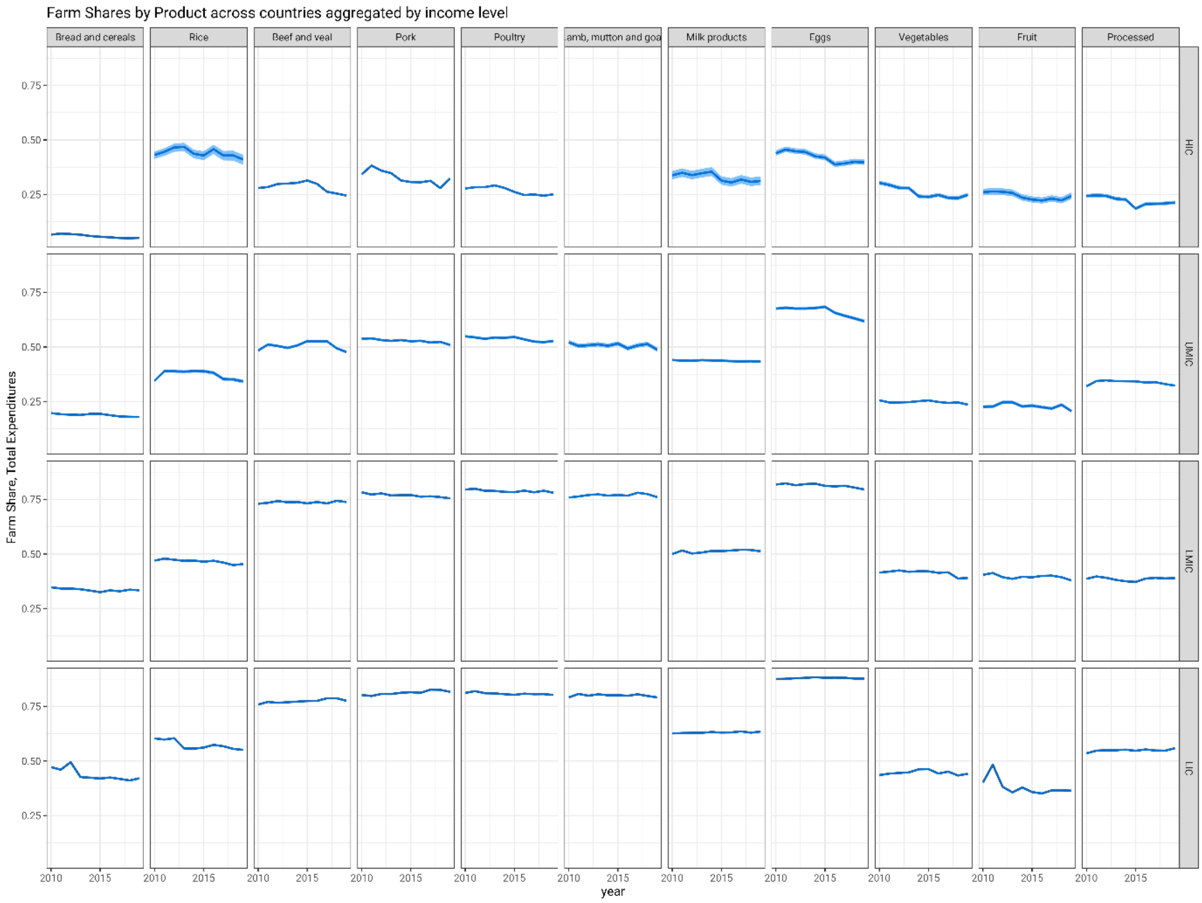

Supplementary Figure 5. Farm shares based on total food expenditures by food product group and country income groups based on World Bank categories. HIC: High-Income Countries, UMIC: Upper-Middle Income Countries, LMIC: Lower-Middle Income Countries, LIC: Low-Income Countries. Blue line model-based estimate of the farm share, shading 95% Credible Interval.

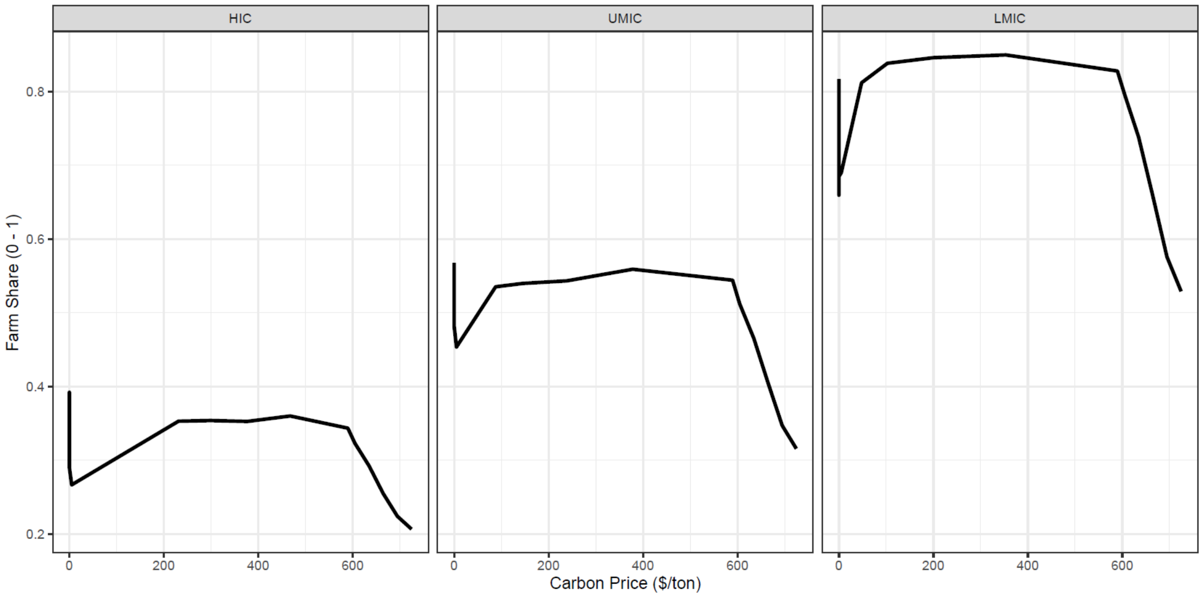

Supplementary Figure 6. Carbon tax vs Farm share for High-Income, Upper-middle, and Lower-Income countries based on World Bank definitions. As carbon tax increases over time, the decline in the farm share is periodically reversed, leading to a flattening, before then decreasing again as margins grow faster than carbon taxes in the latter half of the century.

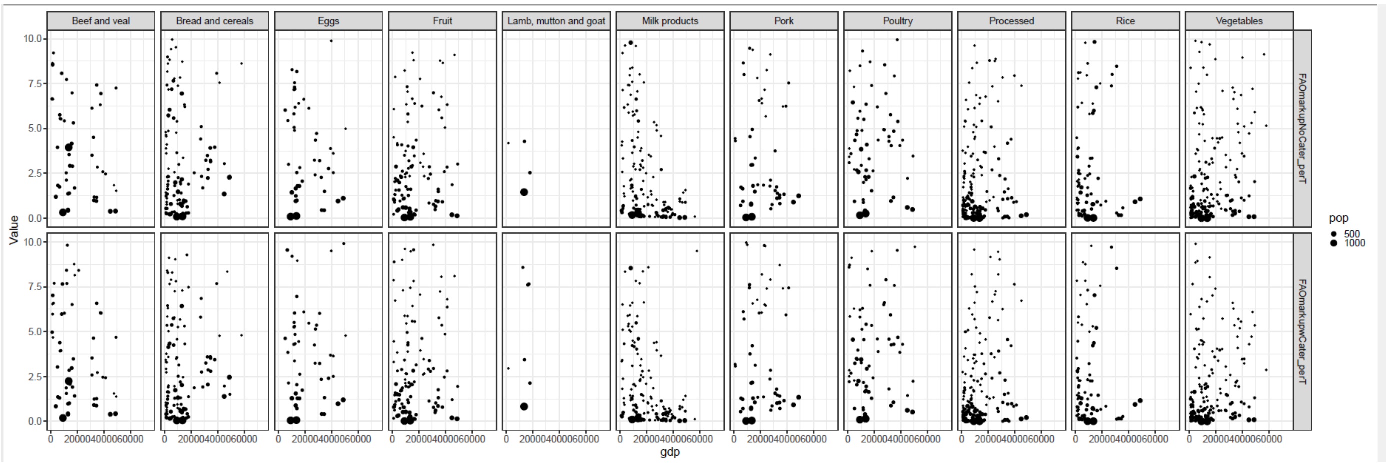

Supplementary Figure 7. Marketing margin calculated on a money-metric (value-based) plotted against GDP per capita.

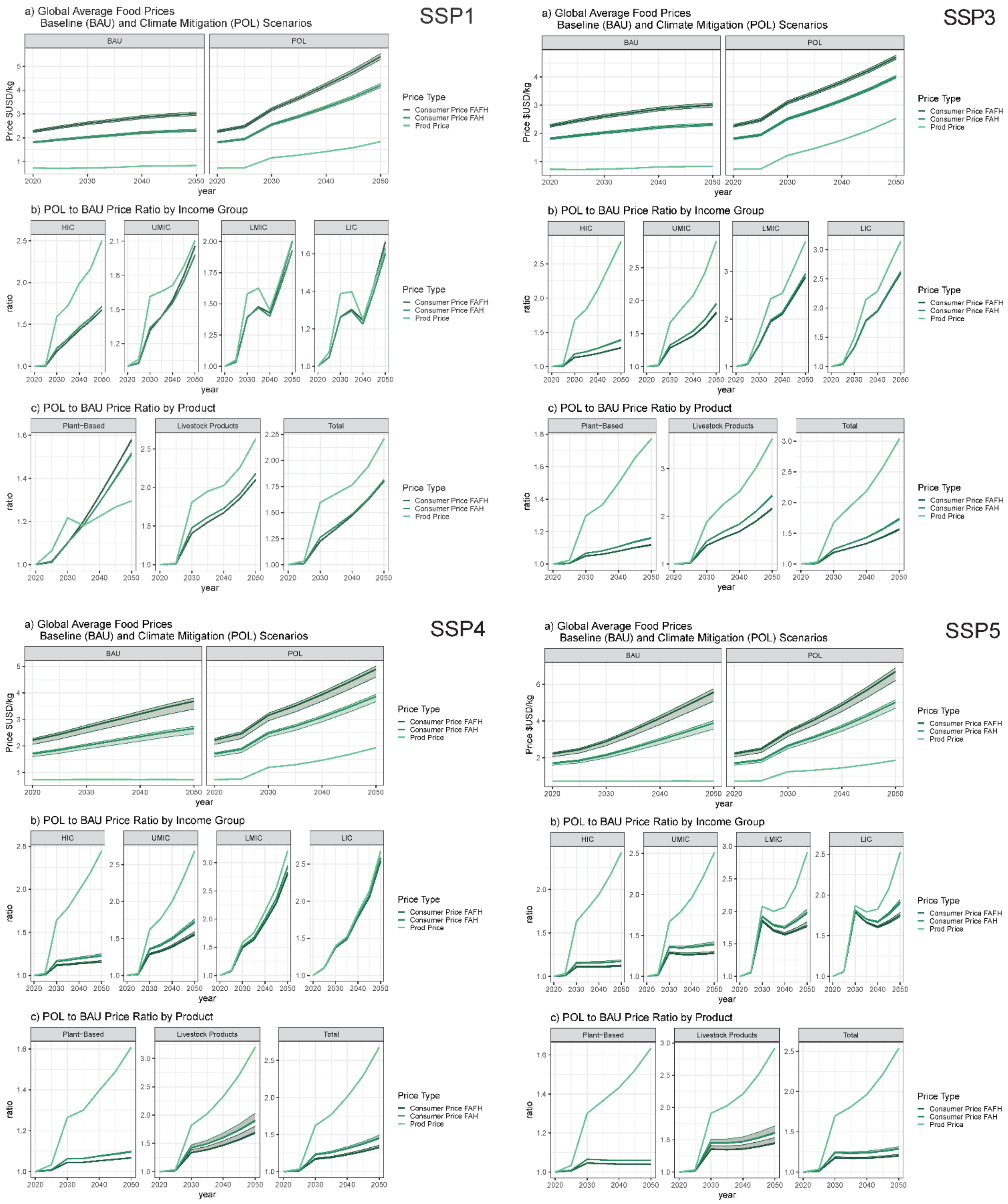

Supplementary Figure 8. Analogous to main text Figure 5, across the 5 SSP scenarios. a) Average food prices in $USD/kg for Business-As-Usual BAU and Climate Mitigation Policy (POL) scenarios, b) Relative price changes as ratio of POL to BAU prices, differentiated across aggregated food groups, and c) regions of the world based on World Bank Income Regions (High, Upper-Middle, Lower-Middle, Lower Income Countries). Prices aggregated by consumption across products and countries, shading represents 95 % Credible Interval of regression results propagated.

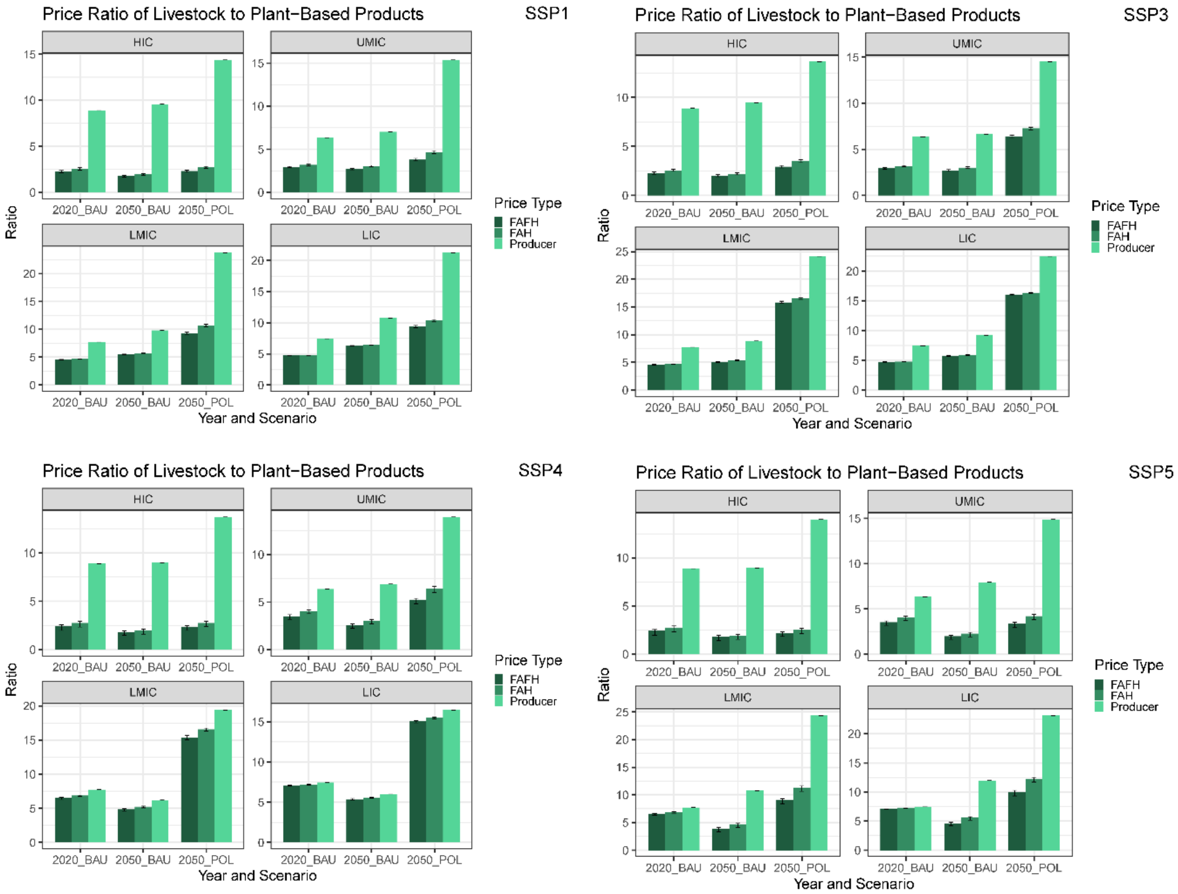

Supplementary Figure 9. Analogous to main text Figure 6, across the 5 SSP scenarios. Price Ratio of Livestock- to Plant-Based Products (aggregated based on tonnes DM consumed) for 2020 (2020_BAU) and 2050 BAU (2050_BAU) and POL (2050_POL) scenarios , across High-Income (HIC), Upper-Middle Income (UMIC), Lower-Middle Income (LMIC) and Low-Income (LIC) Countries, as defined by the World Bank. Error bars represent 95 % Credible Interval of regression results propagated, *n* = 249 countries distributed across income groups.

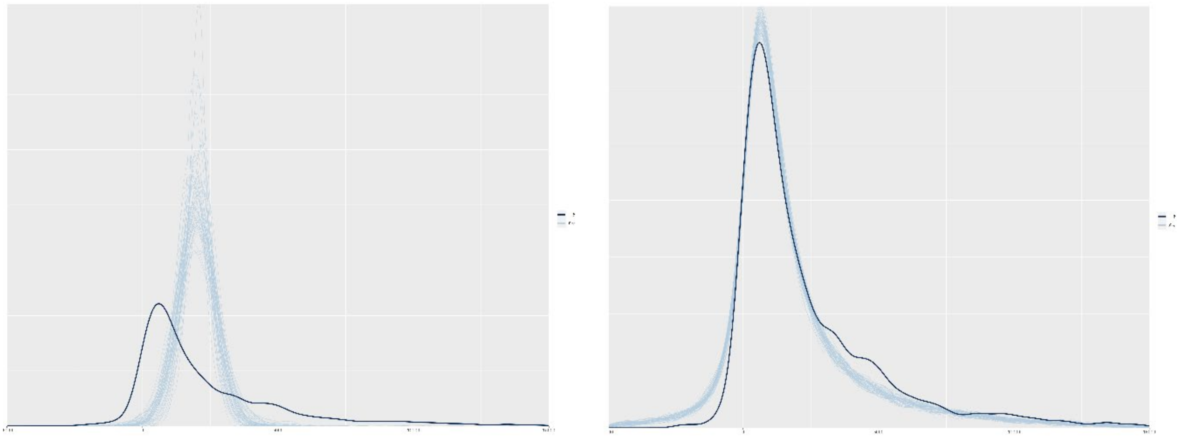

Supplementary Figure 10. Prior and posterior predictive checks. Solid line (y) is data, light lines (y_{rep}) are draws from the a) prior and b) posterior distributions, using 50 draws.

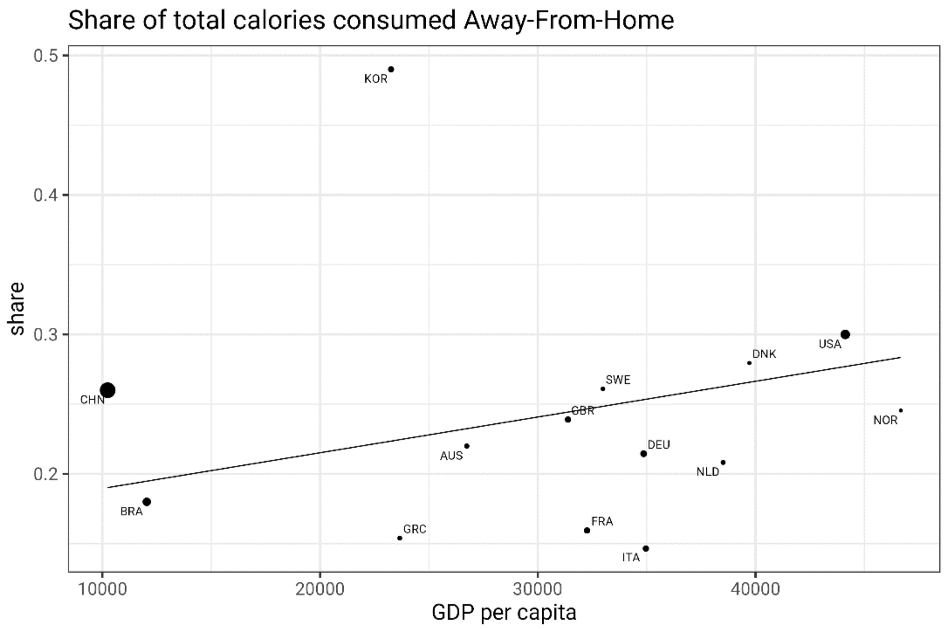

Supplementary Figure 11. Share of total calories consumed away-from-home, with linear regression fit. $y = 1.045e\text{-}01 + 5.603e\text{-}06x$, t-test regression coefficient greater than 0, p = 0.0396, $R^2 = 0.39$.
