# Supplementary material for: Future food prices will become less sensitive to agricultural market prices and mitigation costs
Source: Nat Food. 2025 Jan 3;6(1):85–96. doi: 10.1038/s43016-024-01099-3 (PMC11772223; doi:10.1038/s43016-024-01099-3)
Supplement: Supplementary file 2 — Reporting Summary [file 43016_2024_1099_MOESM2_ESM.pdf]

nature portfolio

Corresponding author(s): David Meng-Chuen Chen

Last updated by author(s): Nov 19, 2024

Reporting Summary

Nature Portfolio wishes to improve the reproducibility of the work that we publish. This form provides structure for consistency and transparency in reporting. For further information on Nature Portfolio policies, see our Editorial Policies and the Editorial Policy Checklist.

Statistics

For all statistical analyses, confirm that the following items are present in the figure legend, table legend, main text, or Methods section.

n/a	Confirmed	
☐	☒	The exact sample size (*n*) for each experimental group/condition, given as a discrete number and unit of measurement
☒	☐	A statement on whether measurements were taken from distinct samples or whether the same sample was measured repeatedly
☐	☒	The statistical test(s) used AND whether they are one- or two-sided<br>*Only common tests should be described solely by name; describe more complex techniques in the Methods section.*
☐	☒	A description of all covariates tested
☐	☒	A description of any assumptions or corrections, such as tests of normality and adjustment for multiple comparisons
☐	☒	A full description of the statistical parameters including central tendency (e.g. means) or other basic estimates (e.g. regression coefficient) AND variation (e.g. standard deviation) or associated estimates of uncertainty (e.g. confidence intervals)
☐	☒	For null hypothesis testing, the test statistic (e.g. *F*, *t*, *r*) with confidence intervals, effect sizes, degrees of freedom and *P* value noted<br>*Give P values as exact values whenever suitable.*
☐	☒	For Bayesian analysis, information on the choice of priors and Markov chain Monte Carlo settings
☐	☒	For hierarchical and complex designs, identification of the appropriate level for tests and full reporting of outcomes
☐	☒	Estimates of effect sizes (e.g. Cohen's *d*, Pearson's *r*), indicating how they were calculated

Our web collection on statistics for biologists contains articles on many of the points above.

Software and code

Policy information about availability of computer code

Data collection	Data was processed and calculated in R version 4.1.2.
Data analysis	The markup data calculated from the ICP and FAOSTAT data was calculated in R, version 4.1.2. Initial data processing and analysis i.e. calculation of markups were conducted via a custom R-package, archived through GitHub here: https://zenodo.org/records/12926687 Output analysis and replication scripts, including MAgPIE output folders are archived at https://zenodo.org/records/12927368. All publically available datasets are archived within the R package; A disaggregated ICP dataset is under confidentiality agreement, and may be made available on a case-by-case basis after communication with both the authors and the ICP.<br>The statistical analysis was conducted via the "brms" R package, version 2.19.0, which is an interface to C++ compiler and Bayesian inference engine "Stan", version 2.21.0. Currency conversion was done with R package "GDPuc" version 3.1.3<br>The MAgPIE-model scenario-based analysis in this paper are computed with the open-source framework MAgPIE (model version MAgPIE 4.7.2 (https://github.com/magpiemodel/magpie/releases/tag/v4.7.2).

For manuscripts utilizing custom algorithms or software that are central to the research but not yet described in published literature, software must be made available to editors and reviewers. We strongly encourage code deposition in a community repository (e.g. GitHub). See the Nature Portfolio guidelines for submitting code & software for further information.

Data

Policy information about availability of data

All manuscripts must include a data availability statement. This statement should provide the following information, where applicable:

- Accession codes, unique identifiers, or web links for publicly available datasets
- A description of any restrictions on data availability
- For clinical datasets or third party data, please ensure that the statement adheres to our policy

Data on producer prices are publicly available online here: https://www.fao.org/faostat/en/#data/PP. The consumer expenditure data is available at coarser aggregation here: https://databank.worldbank.org/source/icp-2017. This study had access to more disaggregate data upon which we are under confidentiality agreement with the ICP; The data is available from the ICP upon request. All further datasets and mappings used are archived within the custom R package: https://zenodo.org/records/12822636. Initial data processing and analysis i.e. calculation of marketing margins were conducted via a custom R-package, archived through GitHub here: https://zenodo.org/record/7963021. Output analysis and replication scripts, including MAgPIE output folders are archived at https://zenodo.org/record/7963068. All publically available datasets are archived within the R package.

Human research participants

Policy information about studies involving human research participants and Sex and Gender in Research.

Reporting on sex and gender	N/A
Population characteristics	N/A
Recruitment	N/A
Ethics oversight	N/A

Note that full information on the approval of the study protocol must also be provided in the manuscript.

Field-specific reporting

Please select the one below that is the best fit for your research. If you are not sure, read the appropriate sections before making your selection.

☐ Life sciences ☐ Behavioural & social sciences ☒ Ecological, evolutionary & environmental sciences

For a reference copy of the document with all sections, see nature.com/documents/nr-reporting-summary-flat.pdf

Ecological, evolutionary & environmental sciences study design

All studies must disclose on these points even when the disclosure is negative.

Study description	Statistical and process-based model analysis of "consumer price markups" on food commodities. Statistical model based on relationship between consumer price markup and per capita income, along with grouping variables of food group and location of consumption.
Research sample	Statistical model based on dataset of 3410 country-year combinations, for the years 2011 and 2017, 11 food groups, and 2 locations of consumption.
Sampling strategy	No new data collected.
Data collection	No new data collected, calculation based on data collected by FAOSTAT and the ICP project associated with the World Bank.
Timing and spatial scale	Statistical model based on years 2011 and 2017, process-based model from 1995-2050.
Data exclusions	Expenditures and demand for alcohol was categorically excluded due to the different marketing and consumption characteristics of this product.
Reproducibility	Scripts for data analysis, statistical and MAgPIE model-run outputs are publically archived online before any publication of manuscript. Source data from the ICP is under confidentiality agreement, full reproducibility (i.e. calculation of markups from source data) will require request to the ICP with discussion from the authours
Randomization	No randomization strategy applied.
Blinding	No new data collected.

Did the study involve field work? ☐ Yes ☒ No

Reporting for specific materials, systems and methods

We require information from authors about some types of materials, experimental systems and methods used in many studies. Here, indicate whether each material, system or method listed is relevant to your study. If you are not sure if a list item applies to your research, read the appropriate section before selecting a response.

Materials & experimental systems

n/a	Involved in the study
☒	☐ Antibodies
☒	☐ Eukaryotic cell lines
☒	☐ Palaeontology and archaeology
☒	☐ Animals and other organisms
☒	☐ Clinical data
☒	☐ Dual use research of concern

Methods

n/a	Involved in the study
☒	☐ ChIP-seq
☒	☐ Flow cytometry
☒	☐ MRI-based neuroimaging
